# Anaemia and Other Haemogram Parameters Associated With Benign Maxillomandibular Odontogenic Lesions

**DOI:** 10.1155/ah/7414742

**Published:** 2025-07-29

**Authors:** Mamadou Diatta, Macoura Gadji, Marie Joseph Diémé, Abdoulaye Keita, Abdou Ba, Bintou Catherine Gassama, Mouhammad Kane, Khadim Seck, Babacar Tamba, Soukeye Dia Tine

**Affiliations:** ^1^Oral Surgery Service, Institute of Odontology and Stomatology, Cheikh Anta Diop University of Dakar (UCAD), Dakar, Senegal; ^2^Odontostomatology Service of Idrissa Pouye General Hospital in Dakar, Dakar, Senegal; ^3^Service of Biological Haematology & Oncology-Haematology (BHOH), Faculty of Medecine, Pharmacy and Odonto-Stomatology (FMPOS), Cheikh Anta Diop University of Dakar (UCAD), Dakar, Senegal; ^4^Department of Medicine, Pathological Anatomy Service, Cheikh Anta Diop University of Dakar (UCAD), Dakar, Senegal

**Keywords:** anaemia, benign odontogenic tumours, haemogram, inflammation, odontogenic cysts

## Abstract

**Introduction:** Dental alterations associated with benign odontogenic lesions can alter patients' diet, possibly leading to anaemia. Indeed, cytological studies of fluid contained in benign odontogenic lesions report the presence of blood cells. The aim of this study was, therefore, to investigate anaemia and haemogram parameters in relation to benign odontogenic lesions.

**Material and Method:** We conducted a descriptive cross-sectional study over 24 months in the Odontostomatology Department of the Idrissa Pouye General Hospital in Dakar, Senegal. The selection criteria included all patients who had received treatment for benign odontogenic lesions with an available cell blood count. The collected variables were demographic, clinical and paraclinical, with calculation of inflammation marker ratios. The data were analysed using SPSS 20.0 software, and the Kruskal–Wallis and Fisher tests were also performed for statistical comparison.

**Results:** Of a total of 50 patients, 70% were women. The mean age was 32.6 years, with a mean duration of 41.5 months. Mandibular location was encountered in 76% of the cases. Ameloblastoma and cemento-osseous dysplasia each accounted for 24% of the cases. Anaemia was found in 21 patients, 11 of whom were normocytic normochromic and 8 microcytic hypochromic. Neutropenia was noted in 23 patients.

**Conclusion:** Normocytic normochromic anaemia, microcytic hypochromic anaemia and neutropenia were more common in benign odontogenic lesions. A more detailed study should be undertaken to gain a better understanding of the significance of haemogram parameter variations in benign odontogenic lesions.

## 1. Introduction

Maxillomandibular odontogenic lesions develop from dental bud cells [1, 2]. Benign lesions, which are more common, include pseudo tumours (cysts) and odontogenic tumours [3, 4]. They account for more than 20% of pathologies affecting the maxillomandibular bone bases [5]. These lesions develop slowly, progressively and sometimes silently, requiring consultation only when pain, functional and/or aesthetic discomfort appear [4, 6]. Some of these lesions lead to bone lysis, sometimes containing a liquid whose cytological study reveals the presence of red blood cells [7, 8]. In addition, the neovascularisation that is essential for the proliferation of cancerous cells, combined with bone and tooth lysis, leads to displacement, mobility and/or loss of teeth. This later will affect mastication, thus disrupting patients' diet, with possible repercussions on their general condition [9, 10]. According to some authors [11–13], there is also a relationship between anaemia and dentoparodontal lesions; they have reported that the inflammatory phenomena encountered during chronic generalised periodontitis lead to anaemia and an increase in leucocyte and lymphocyte counts, MCV values and RDW [11–13]. Odontogenic lesions have also been reported in patients with general pathologies leading to anaemia, such as sickle cell disease [14].

This relationship between benign odontogenic lesions and anaemia prompted the initiation of this study, aiming to investigate anaemia and other biological variables of the haemogram during the course of benign maxillomandibular odontogenic lesions.

## 2. Materials and Methods

We conducted a descriptive cross-sectional study of patients with benign odontogenic lesions managed at the Odontostomatology Department of the Idrissa Pouye General Hospital in Dakar, Senegal. Sampling was exhaustive, with the selection of all patients who presented with a benign maxillomandibular odontogenic lesion after free and informed consent. This study was approved by the Ethics Committee of Cheikh Anta Diop University in Dakar (026612017/ASS/CER/UCAD). The selection criteria were all patients who had their benign odontogenic maxillomandibular lesion treated in the department, under local or general anaesthesia, with a cell blood count and regular follow-up. The collected variables include epidemiological aspects (age and sex), clinical aspects (duration of evolution, location of the lesion, etc.) and paraclinical aspects (haemogram, nosological group and histological type). These variables were recorded in a data collection form over a 24-month period. A total of 7 other variables were calculated, representing indices and good markers of inflammation [11, 15, 16]. These were platelet to red blood cell ratio (PGGR) ∗ 1000, red blood cell to platelet ratio (GRPR) ∗ 1000, neutrophil to lymphocyte ratio (NLR) ∗ 1000, neutrophil to platelet ratio (NPR) ∗ 1000, neutrophil to monocyte ratio (NMR) ∗ 1000, monocyte to lymphocyte ratio (MLR) ∗ 1000 and platelet to lymphocyte ratio (PLR) ∗ 1000 [11, 15, 16].

The data were entered into a Microsoft Excel 13 spreadsheet and analysed using SPSS 20 on Windows. Data were expressed as frequencies and means with standard deviations after verification of the Gaussian distribution for quantitative variables. As the variables did not follow the normal distribution, the Kruskal–Wallis test was used to determine their distribution according to histological type or nosological group. Finally, the Fisher test was used to assess the association between certain variables.

## 3. Results

### 3.1. Sociodemographic Aspects

Women represented 70% (*n* = 35) of the studied population with a sex ratio of 0.43 (Table 1).

The mean age was 32.6 ± 17.8 years, with a median of 27.5 years and extremes of 7 and 76 years. The 16–30 age group represented 34% (*n* = 17) of the studied population, the 31–45 age group 22% (*n* = 11) and the 1–15 age group 20% (Table 1).

### 3.2. Clinical Aspects

The duration mean of the disease was 41.5 ± 25.5 months, with a median of 36 months and extremes of 18 and 120 months. Progression time between 24 and 59 months was noted in 56% (*n* = 28) of the patients (Table 1).

Mandibular location was the most represented with 76% (*n* = 38) of the patients (Table 1).

### 3.3. Paraclinical Aspects

#### 3.3.1. Histological Types

Ameloblastomas and cemento-osseous dysplasias, both represented each 24% (*n* = 12) of the patients, inflammatory cysts 22% (*n* = 11) and dentigerous cysts 18% (*n* = 9) (Table 2).

#### 3.3.2. Nosological Groups

**Figure 1:**
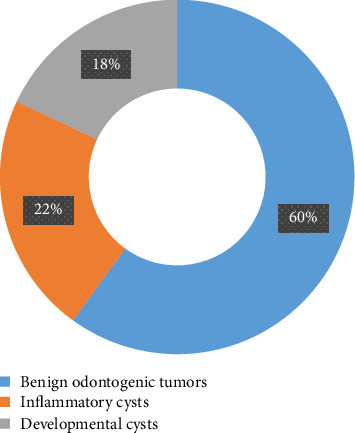
Distribution of the study population according to nosological group of lesions.

Benign odontogenic tumours were found in 60% (*n* = 30) of the patients, inflammatory cysts in 22% (*n* = 11) and developmental cysts in 18% (*n* = 9) (Figure 1).

#### 3.3.3. Haemogram

Analysis of the red blood cell line (erythrocyte count, haemoglobin level and erythrocytic indices) revealed anaemia in 21 patients (42%), including 11 cases of normocytic normochromic anaemia, 8 cases of microcytic hypochromic anaemia and 2 cases of normocytic hypochromic anaemia. In addition, a decrease in mean corpuscular haemoglobin content (MCHC) was noted in 14 patients (28%), a decrease in RBC count in 7 patients (14%), a decrease in MCHC in 5 patients (10%) and anisocytosis in 4 patients (8%).

Analysis of the platelet lineage revealed thrombocytosis (hyperplaquettosis) in 4 patients (8%) and thrombocytopaenia in 1 patient (2%).

Analysis of the leucocyte counts and repartition described neutropenia in 23 patients (46%) and lymphopenia in 7 patients (14%). It was also noted an increase in the count of eosinophilic polymorphonuclear cells in 7 patients (14%) (Table 3).

### 3.4. Age Groups According to MCHC

Of the 5 patients with a decreased MCHC value, 4 (80%) had a benign odontogenic tumour with a significant difference (*p* value = 0.040) (Table 4).

Furthermore, the type of anaemia was not associated with the nosological groups of benign odontogenic lesions.

### 3.5. Nosological Groups According to Haematocrit (HCT)

Of the 12 patients with a reduced HCT value, 50% were found in developmental cysts with a *p* value < 0.05 (Table 5).

### 3.6. Platelets/Red Blood Cells Ratio ((PRBCR) ∗ 1000) According to Histological Types of Lesions

The means for odontogenic keratocysts, dentigerous cysts and complex odontoma are higher with a significant difference (*p*=0.002) (Table 6).

### 3.7. Platelets/Red Blood Cells Ratio ((PRBCR) ∗ 1000) According to Nosological Groups of Lesions

The means for developmental cysts and benign odontogenic tumours are higher with a significant difference (*p*=0.047) (Table 7).

The associations sought between certain ratios and histological types and nosological groups of benign odontogenic lesions were not significant (Appendices: Tables A1, A2, A3, A4, A5 and A6).

## 4. Discussion

Benign odontogenic lesions are highly polymorphic lesions, in which several are easy to diagnose but others are difficult to diagnose because of their similar clinical and/or radiological features. A positive diagnosis is often confirmed after pathological examinations. That is why some biological parameters, such as haemograms, could make a major contribution to the diagnosis and management of these lesions.

### 4.1. Sociodemographic Aspects

In this present study, a predominance of females (70%) was noted, with a sex ratio of 0.43. These results are similar to those reported by certain authors with a female predominance in benign maxillomandibular odontogenic lesions [5, 10]. However, other studies have described a male predominance [1, 17]. This difference could be explained by the small sample size in this study. These lesions can occur at any age [10]. In addition, the affected population was young, with an average age of 32.6 years and a predominance of the 16–30 age group (34%). These results are similar to those reported by Kaur et al. 2021 [1], with an average age of 31.9 years. However, higher mean ages have been reported by other authors [10, 18] on a larger sample size. Besides the sociodemographic aspects, clinical symptoms were also studied.

### 4.2. Clinical Symptoms

The duration of odontogenic lesions varies from a few months to several years [10, 17]. These lesions are sometimes discovered by chance during a routine radiological examination [19]. The mean duration of evolution was 41.5 months, with more than 50% of cases having a duration of evolution in between 24 and 59 months. These results differ from those reported by Sayela et al. in 2020, with 52.8% of the patients having a duration of less than 24 months [10]. In addition, shorter durations of progression were also reported [10, 20]. This difference could be explained by poverty, ignorance, societal beliefs, reliance on traditional practitioners and lack of infrastructure [20].

Benign odontogenic lesions can be located in the maxilla as well as the mandible. The mandible was the most frequently reported site in the literature, accounting for more than 60% of the cases [1, 5, 10]. This is in perfect agreement with the results of this present study, in which the mandibular location was found in 76% of the patients.

### 4.3. Paraclinical Aspects

According to the literature, ameloblastoma, with its various histological forms, is the most frequently encountered benign odontogenic maxillomandibular tumour [1, 18]. In fact, the high number of cases of cemento-osseous dysplasia (24%) found in this study could be explained by the frequency of this lesion, which is most often found in the elderly, particularly women, in Senegal [21]. Ameloblastomas are locally aggressive lesions prompted to recur. They are clinically manifested by deformation of the bone tables, displacement and mobility of the teeth, thus impairing the patient's quality of life. On radiography, the image may be unigeodic radiolucent or multigeodic (classic forms) with rhizalysis of the dental roots.

Other lesions such as inflammatory cysts have also been reported by some authors [5, 22]. Inflammatory cysts are chronic lesions of the periodontal tissue resulting from bacterial infection of the endodontium or periodontium, which, as they develop, lead to deformation of the bony bases and may even break out after rupture of the bony cortex.

Cemento-osseous dysplasias are fibro-osseous lesions associated with dental apices, which develop slowly and silently. They may be discovered by chance during a radiographic examination. In florid forms, the radiograph shows mixed images (osteolytic and osteocondensing) poorly limited around the apices of several teeth. The lesion may be painful if superinfected. In focal and florid clinical forms of cemento-osseous dysplasia, the lesions are often surrounded by granulation tissue, which is highly haemorrhagic during surgical removal.

These classical clinical manifestations might impair the patient nutrition and lead to nutritional anaemia. Thus, besides the clinical and paraclinical aspects, study of benign maxillomandibular odontogenic lesions requires the use of complementary examinations such as haemogram.

### 4.4. Cell Blood Counts (Haemograms)

Benign maxillomandibular odontogenic lesions, as they progress, will lead to bone deformations associated with displacements, retentions, mobility or loss of teeth, which may impair the patient's mastication, with a possible impact on the general state of health and thus on certain cell blood count parameters, including the haemoglobin level. In this present study, anaemia was found in 21 patients (42%), including 11 cases of normocytic normochromic anaemia, 8 cases of microcytic hypochromic anaemia and 2 cases of normocytic hypochromic anaemia. According to the literature, microcytic anaemia is most often hypochromic with variable aetiologies that may be related to nutritional deficiency in addition to inflammatory phenomena [23, 24]. According to Lanier et al. 2021, anaemia in adults may be asymptomatic and discovered fortuitously [25]. The most commonly reported causes are nutritional deficiencies and/or insufficiencies, which may be encountered in patients with benign odontogenic lesions due to impaired mastication [9, 10]. In addition, as odontogenic lesions progress, they create neovascularisation to satisfy the need of blood nutriments, which is essential for the proliferation and development of cancerous cells [26]. According to Fatemeh et al., 2017 [27], the cytological study of puncture fluids of benign odontogenic lesions reports the presence of red blood cells.

Moreover, the 2 cases of normocytic hypochromic anaemia encountered could be an intermediate phase due either to an iron deficiency in the body or to a defect in iron utilisation due to the presence of inflammatory phenomena [28].

A decrease in HCT values and mean corpuscular haemoglobin concentration was associated with developmental cysts, which could be explained by the blood cell requirements of these lesions, particularly neoangiogenesis, for their growth and development [26].

Thrombocytosis was associated with the 1–15 year age group and with a duration of evolution of 1–23 months. In contrast, thrombocytopaenia was associated with the age range of 60 years and later as well as with a duration of evolution of 60 months and above. According to Sharma et al., 2013 [6], besides their involvement in haemostasis, platelets are thought to mediate the growth, dissemination and angiogenesis of tumour lesion cells [6]. The association of thrombocytopaenia with advanced age could be explained by a decrease in platelet levels reported beyond the age of 60 [29].

The anisocytosis associated with cemento-osseous dysplasia could be explained by the fact that these lesions are often surrounded by highly haemorrhagic granulation tissue due to chronic infiltration of inflammatory cells into the lesion site [30]. These chronic inflammatory phenomena promote the supply of blood cells to the site, which is essential for the cellular repair reaction. The histological appearance of cemento-osseous dysplasia shows the presence of small blood-filled vessels in the stroma, which may explain the influx of blood cells into the bone tissue. In the initial stage, these lesions consist of unencapsulated fibrous connective tissue with numerous small blood vessels [31]. According to Urs et al. [32], histological sections of cemento-osseous dysplasia sometimes show haemorrhagic areas.

Furthermore, the statistically significant associations observed between high mean PRBCR ratios in developmental cysts (dental cysts) and benign odontogenic tumours (odontogenic keratocysts and complex odontoma) might be explained by the fact that platelets are essential for the development and progression of tumour cells in certain types of lesion [6].

## 5. Conclusion

Benign odontogenic lesions are diverse and varied, with different clinical and radiological manifestations. They may affect the patient's mastication, with possible repercussions on patient nutrition and his general condition. Neutropenia and anaemia were the most common abnormalities of haemogram parameters. Anaemia as normocytic normochromic anaemia was mostly recorded, followed by microcytic hypochromic anaemia and finally by fewer normocytic hypochromic anaemia. Thus, haemogram appears to be very useful for stratification and follow-up of benign odontogenic lesions. However, a more inclusive study should be undertaken to gain a better understanding of the variations in haemogram parameters and especially anaemia in benign maxillomandibular odontogenic lesions.

## Figures and Tables

**Table 1:** Distribution of the study population by sex, location and time interval of lesion evolution.

Variables		Number (*n*)	Percentage (%)	Total *n* (%)
Gender	Men	15	30	50 (100)
	Women	35	70	

Age 15 years and over	(1–15) years	10	20	50 (100)
	(16–30) years	17	34	
	(31–45) years	11	22	
	(46–60) years	9	18	
	> 60 years	3	6	

Localization	Maxillary	12	24	50 (100)
	Mandible	38	76	

Duration of evolution in months	(1–24) months	11	22	50 (100)
	(24–60) months	28	56	
	≥ 60 months	11	22	

**Table 2:** Distribution of study population according to histological aspects of lesions.

Histological types	Numbers (*n*)	Percentage (%)
Ameloblastoma	12	24
Cemento-bone dysplasia	12	24
Inflammatory cyst	11	22
Dentigerous cyst	9	18
Odontogene keratocyst	5	10
Complex odontoma	1	2
Total	50	100

**Table 3:** Distribution of the study population according to variation in haemogram parameters.

Blood count parameters	Variation in haemogram parameters			Total *n* (%)
	Decreased (means ± standard deviations)	Normal (means ± standard deviations)	Increased (means ± standard deviations)	
Red blood cells, *n* (%)	(3,552,857.14 ± 1,038,728.62/mm^3^) 7 (14)	(4,708,333.33 ± 457,314.09/mm^3^) 42 (84)	(5,570,000/mm^3^) 1 (2)	50 (100)
Haemoglobin, *n* (%)	(11.22 ± 0.76 g/dL) 21 (42)	(13.36 ± 1.29 g/dL) 29 (58)	—	50 (100)
Haematocrit, *n* (%)	(33.12 ± 1.79%) 12 (24)	(39.92 ± 4.03%) 38 (76)	—	50 (100)
Mean corpuscular volume, *n* (%)	(72.85 ± 3.04 fL) 8 (16)	(83.76 ± 4.51 fL) 37 (74)	(95,94 ± 3,01 fL) 5 (10)	50 (100)
Mean corpuscular haemoglobin content, *n* (%)	(24.24 ± 2.58 pg) 14 (28)	(28.05 ± 1.74 pg) 35 (70)	(34.70 pg) 1 (2)	50 (100)
Mean corpuscular haemoglobin concentration, *n* (%)	(28.48 ± 1.64 g/dL) 5 (10)	(33.05 ± 1.68 g/dL) 45 (90)	—	50 (100)
RDW-CV, *n* (%)	—	(13.63 ± 1.15%) 46 (92)	(19.07 ± 1.37%) 4 (8)	50 (100)
Platelets, *n* (%)	(142,000/mm^3^) 1 (2)	(300,133.33 ± 74,163.70/mm^3^) 45 (90)	(475,500.00 ± 30,512.29/mm^3^) 4 (8)	50 (100)
White blood cells, *n* (%)	(2250/mm^3^) 1 (2)	(5879.59 ± 1575.40/mm^3^) 49 (98)	—	50 (100)
Lymphocytes, *n* (%)	(1286.43 ± 192.80/mm^3^) 7 (14)	(2454.50 ± 850.45/mm^3^) 42 (84)	(5250/mm^3^) 1 (2)	50 (100)
Neutrophils, *n* (%)	(1822.61 ± 410.97/mm^3^) 23 (46)	(3312.58 ± 832.38/mm^3^) 25 (50)	(4650.00 ± 636.40/mm^3^) 2 (4)	50 (100)
Monocytes, *n* (%)	(40/mm^3^) 2 (4)	(532.91 ± 181.28/mm^3^) 47 (94)	(1600/mm^3^) 1 (2)	50 (100)
Eosinophilic polynuclear cells, *n* (%)	—	(156.95 ± 117.70/mm^3^) 43 (86)	(1198.57 ± 373.92/mm^3^) 7 (14)	50 (100)
Basophilic polynuclear cells, *n* (%)	—	(33.8 ± 23.8/mm^3^) 50 (100)	—	50 (100)

**Table 4:** Nosological groups according to mean corpuscular haemoglobin concentration (MCHC) value.

Nosological groups	MCHC (g/dL)		Total *n* (%)	*p* value
	Decreased (28.70; Min.: 26.60, Max.: 30.60) *n* (%)	Normal (33.05 ± 1.68 g/dL) *n* (%)		
Developmental cysts	—	9 (20)	9 (18)	0.040
Inflammatory cysts	1 (20)	10 (22.2)	11 (22)	
Benign odontogenic tumors	4 (80)	26 (57.8)	30 (60)	
Total	5 (100)	45 (100)	50 (100)	

**Table 5:** Distribution of lesion type according to haematocrit (HCT).

Nosological groups	HCT (%)		Total *n* (%)	*p* value
	Decreased (33.60; Min.: 28.70, Max.: 35.20) *n* (%)	Normal (39.92 ± 4.03) *n* (%)		
Developmental cysts	5 (41.7)	4 (10.5)	9 (18)	0.007
Inflammatory cysts	1 (8.3)	10 (26.3)	11 (22)	
Benign odontogenic tumours	6 (50)	24 (63.2)	30 (60)	
Total	12 (100)	38 (100)	50 (100)	

**Table 6:** Platelets/red blood cells ratio (PRBCR) ∗ 1000) according to histological types of lesions.

Histological types	PRBCR		
	Numbers (*n*)	Averages	Interquartile range
Ameloblastoma	12	70,89,311	25,23,161
Cemento-bone dysplasia	12	65,34,028	15,26,703
Inflammatory cyst	11	55,84,149	17,00,843
Dentigerous cyst	9	79,25,008	20,12,271
Odontogenic keratocyst	5	103,356	64,89,779
Complex odontoma	1	94,97,816	—
Total	50	71,15,537	30,07,235
*p* value	0.002		

**Table 7:** Platelets/red blood cells ratio (PRBCR) ∗ 1000) according to nosological groups of lesions.

Nosological groups	PRBCR		
	Numbers (*n*)	Averages	Interquartile range
Developmental cysts	9	77,31,463	26,30,215
Inflammatory cysts	11	55,94,345	16,13,579
Benign odontogenic tumours	30	74,88,529	33,66,708
Total	50	71,15,537	30,07,235
*p* value	0.047		

**Table 8:** Neutrophil/lymphocyte ratio (NLR), neutrophil/platelet ratio (NPR) and neutrophil/monocyte ratio (NMR) according to the histological type.

Histological types	NLR			NPR			NMR		
	Numbers (*n*)	Averages	Interquartile range	Numbers (*n*)	Averages	Interquartile range	Numbers (*n*)	Averages	Interquartile range
Ameloblastoma	12	1,344,673	1,314,501	12	8,758,669	8,583,863	12	5,498,694	5,914,021
Cemento-bone dysplasia	12	1,168,701	978,1061	12	8,074,056	31,144	12	3,630,303	1282,41
Inflammatory cyst	11	1,051,620	1,316,086	11	813,417	9,578,755	11	4072,2605	3,136,264
Dentigerous cyst	9	15,125,2725	890,6357	9	9,736,561	48,627,685	9	5,551,141	2,806,9138
Odontogenic keratocyst	5	518,797	600,5747	5	5,287,356	2,832,425	5	7000	5,111,336
Complex odontoma	1	1000	0	1	4,597,701	0	1	2,857,143	0
Total	50	1,070,017	985,337	50	8,238,529	6,101,385	50	4,647,841	3,231,502
*p* value	05,211			03,308			00,721		

**Table 9:** Monocyte to lymphocyte ratio (MLR) and platelet to lymphocyte ratio (PLR) according to the histological type.

Histological types	MLR			PLR		
	Numbers (*n*)	Averages	Interquartile range	Numbers (*n*)	Averages	Interquartile range
Ameloblastoma	12	264,4013	114,8777	12	121720,9	104065,3
Cemento-bone dysplasia	12	260,9579	229,5468	12	126363,6	100558,7
Inflammatory cyst	11	291,6211	111,4799	11	146910,25	43438,29
Dentigerous cyst	9	213.1601	127,36,785	9	151,873,205	54,189,025
Odontogenic keratocyst	5	99,23,664	73,89,163	5	104482,8	14856,8
Complex odontoma	1	350	0	1	217,500	0
Total	50	225,5167	150,3586	50	129931,8	90768,59
*p* value	00,566			02,304		

**Table 10:** Neutrophil/lymphocyte ratio (NLR), neutrophil/platelet ratio (NPR) and neutrophil/monocyte ratio (NMR) according to nosological groups.

Nosological groups	NLR			NPR			NMR		
	Numbers (*n*)	Averages	Interquartile range	Numbers (*n*)	Averages	Interquartile range	Numbers (*n*)	Averages	Interquartile range
Developmental cysts	9	1,269,767	662,3377	9	9,705,882	5,123,562	9	4,714,286	2,332,181
Inflammatory cysts	11	1,035,533	1,316,086	11	6,890,756	9,578,755	11	5250	3,670,655
Benign odontogenic tumours	30	1,033,023	1058.523	30	8,074,056	3.26934	30	4,269,421	3,611,111
Total	50	1,070,017	985,337	50	8,238,529	6,101,385	50	4,647,841	3,231,502
*p* value	05,573			05,230			06,150		

**Table 11:** Monocyte/lymphocyte ratio (MLR) and platelet/lymphocyte ratio (PLR) according to nosological groups.

Histological types	MLR			PLR		
	Numbers (*n*)	Averages	Interquartile range	Numbers (*n*)	Averages	Interquartile range
Developmental cysts	9	213,6364	143,2558	9	149667,9	104293,3
Inflammatory cysts	11	242,0382	102,7778	11	139370,1	43438,29
Benign odontogenic tumours	30	228,5803	178,5714	30	125672,5	90768,59
Total	50	225,5167	150,3586	50	129931,8	90768,59
*p* value	08,711			07,003		

**Table 12:** Red blood cells/platelets (RBCPR) ∗ 1000 according to histological types.

Histological types	PRBCR		
	Numbers (*n*)	Averages	Interquartile range
Ameloblastoma	12	16441,07	7,697,534
Cemento-bone dysplasia	12	16108,24	3,396,782
Inflammatory cyst	11	18684,68	5,813,871
Dentigerous cyst	9	13862,33	3 188,5845
Odontogenic keratocyst	5	11807,34	4,356,485
Complex odontoma	1	10528,74	—
Total	50	16000,68	5,680,012
*p* value	0.087		

**Table 13:** Red blood cells/platelets (RBCPR) ∗ 1000 according to nosological groups of lesions.

Nosological types	PRBCR		
	Numbers (*n*)	Averages	Interquartile range
Developmental cysts	9	14190,47	4,350,505
Inflammatory cysts	11	19287,52	5,533,389
Benign odontogenic tumours	30	15338,57	5,758,905
Total	50	16000,68	5,680,012
*p* value	0.654		

## Data Availability

The data used to support the findings of this study are included within the article and are available from the corresponding author upon reasonable request.

## Appendix A: Cross-Referencing Between Other Ratios and Histological Types or Nosological Groups of Benign Odontogenic Lesions

NLR, NPR and NMR according to the histological type: The medians of the NLR and NMR were higher in dentigerous cysts than those in ameloblastomas, with no significant difference (*p*=0.521 for NLR and *p*=0.33 for NPR) (Table A1).

MLR and PLR according to the histological lesion type: The median MLR ratios are higher in ameloblastomas and cemento-osseous dysplasias than those in dentigerous and inflammatory cysts. The median PLR was higher in dentigerous cysts than that in ameloblastomas and cemento-osseous dysplasias without a significant difference (*p*=0.056 for MLR and *p*=0.230 for PLR) (Table A2).

NLR, NPR and NMR according to nosological groups: The median NLR is higher in developmental cysts than in inflammatory cysts and benign odontogenic tumours. The median NMR was higher in inflammatory cysts than that in developmental cysts and benign odontogenic tumours without a significant difference (*p*=0.557 for NLR and *p*=0.615 for NMR) (Table A3).

MLR and PLR in terms of nosological groups: The median MLR is higher in inflammatory cysts than that in benign odontogenic tumours and developmental cysts. The median PLR was higher in developmental cysts than that in inflammatory cysts and benign odontogenic tumours without a significant difference (*p*=0.871 for MLR and *p*=0.700 for PLR) (Table A4).

RBCPR ∗ 1000 according to histological types: The means for inflammatory cysts, residual cysts, pericoronary cysts and ameloblastomas are higher without a significant difference (*p*=0.087) (Table A5).

RBCPR ∗ 1000 according to nosological groups of lesions: The mean of inflammatory cysts is higher without a significant difference (*p*=0.654) (Table A6).
